# Multi-Horizon Herd-Based Cattle Live-Weight Forecasting Using Irregular Automated Weighing Data

**DOI:** 10.3390/ani16152436

**Published:** 2026-08-06

**Authors:** Muhammad Riaz Hasib Hossain, Rafiqul Islam, Shawn R. McGrath, Md Zahidul Islam, David W. Lamb

**Affiliations:** 1 School of Computing, Mathematics and Engineering, Charles Sturt University, Wagga Wagga, NSW 2650, Australia; 2 School of Computing, Mathematics and Engineering, Charles Sturt University, Albury, NSW 2640, Australia; mislam@csu.edu.au; 3 Gulbali Institute for Agriculture, Water and Environment, Charles Sturt University, Wagga Wagga, NSW 2678, Australia; shmcgrath@csu.edu.au; 4 School of Computing, Mathematics and Engineering, Charles Sturt University, Panorama Avenue, Bathurst, NSW 2795, Australia; zislam@csu.edu.au; 5 AI and Cyber Futures Centre, Charles Sturt University, Panorama Avenue, Bathurst, NSW 2795, Australia; 6 Precision Agriculture Research Group, University of New England, Armidale, NSW 2351, Australia; dave.lamb@foodagility.com; 7 Food Agility CRC Ltd., Sydney, NSW 2000, Australia

**Keywords:** cattle live-weight forecasting, machine learning, gradient boosting, precision livestock farming, automated weighing systems, multiple forecasting periods

## Abstract

Forecasting individual cattle live weight in herd-managed grazing systems remains challenging because automated weighing observations are irregular and environmental conditions vary seasonally. Evidence remains limited for live-weight forecasting across multiple forecasting periods under commercial grazing conditions. This study developed a machine learning (ML) framework for forecasting the live weight of individual cattle using automated observations from a managed herd. The framework incorporated demographic variables, historical live weights, and climatic lag predictors. The framework compared monthly, weekly, and rolling-window aggregations across forecasting periods of 1, 2, and 3 months. Quality control retained 494 of 1140 cattle (43.3%), yielding 4069 monthly aggregated records. The resulting dataset was more suitable for modelling cattle with regular voluntary weighing records. The respective forecasting datasets contained 3048, 2558, and 2068 records for one-, two-, and three-month periods. Gradient Boosting achieved the strongest testing performance. The corresponding coefficients of determination (R^2^) were 0.950, 0.935, and 0.902. Monthly aggregation achieved higher entropy retention, greater variance preservation, and stronger forecasting performance than alternative aggregation approaches. Feature-importance analysis identified animal age and historical live weight as the most important predictors across all forecasting periods. Lagged rainfall and temperature variables provided complementary predictive information for medium-term forecasting. The findings demonstrate that automated livestock monitoring, climatic information, and ML can support accurate live-weight forecasting. The framework produced forecasts across multiple periods for individual cattle in herd-managed grazing systems.

## Simple Summary

Forecasting the live weight of individual cattle in herd-managed grazing systems supports feed management, grazing decisions, and welfare monitoring in commercial beef enterprises. In this study, a herd-based forecasting framework was developed to predict future live weight for individual cattle managed within a commercial grazing herd. The framework integrated automated weighing observations, climatic information, and animal characteristics across multiple forecasting periods. Early identification of abnormal growth patterns may support timely management intervention during nutritional or environmental stress. Forecasting outputs may improve pasture allocation, supplementation planning, and seasonal grazing management within precision livestock farming systems.

## 1. Introduction

Cattle production remains a major component of global food systems and contributes substantially to meat supply, rural livelihoods, and agricultural sustainability [1,2]. Efficient herd growth management strongly influences feed utilisation, production efficiency, economic profitability, and animal welfare in grazing-based beef systems [3]. Climatic variability, pasture instability, and rising production costs have increased demand for accurate forecasting systems [4,5]. Such systems can support proactive grazing management and long-term production planning. Climate-driven pasture fluctuations may substantially alter cattle growth trajectories and reduce forecast reliability across extensive grazing environments [6].

Traditional cattle-weight assessment commonly relies on manual handling, visual estimation, or periodic measurements involving physical restraint [7]. These methods increase labour requirements and introduce observational variability. Repeated physical handling may elevate animal stress and reduce measurement consistency under grazing conditions [8]. Irregular sampling frequency also limits early detection of abnormal growth trajectories and delays management intervention within commercial beef enterprises [9].

Precision livestock farming (PLF) technologies increasingly integrate sensor systems, cloud-based monitoring, and AI-driven analytics to support adaptive livestock management and predictive decision-making [2]. Step-on-off (SOO) weighing systems enable continuous live-weight monitoring during normal cattle movement without intensive manual handling [10]. Systems such as Optiweigh use attractants, including molasses and salt blocks, to encourage animals to visit the weighing station voluntarily [11]. Voluntary animal participation enables frequent, low-stress acquisition of live-weight data under commercial grazing conditions [8]. Continuous livestock monitoring can improve operational efficiency and welfare surveillance [12]. Machine learning (ML) models can transform continuous sensor data from descriptive monitoring into predictive decision support [3]. These models can identify growth patterns, health risks, and production responses from repeated observations [13]. Predictive PLF analytics may then support earlier intervention and improved resource optimisation [13].

Existing livestock prediction studies mainly evaluate body-weight estimation, animal identification, or growth assessment under controlled observational conditions [14]. Many studies use image-based or structured datasets collected under consistent sampling schedules [15]. Commercial grazing systems yield irregular observations because animal interactions with automated infrastructure vary over time and across environmental conditions [16]. Evidence remains limited for individual live-weight forecasting across multiple forecasting periods using irregular automated records collected under commercial grazing conditions [17]. The predictive contribution of climatic lag effects also remains unclear for medium-term cattle growth forecasting in pasture-based systems [18]. Improved biological interpretation of forecasting outputs remains important for supporting management decisions and producer adoption within precision livestock farming systems [19].

Automated in-paddock weighing and related sensor systems provide repeated growth records under practical production conditions [7]. However, many existing weight-estimation studies rely on controlled imaging, dairy settings, or structured observation schedules [20]. Forecasting future live weight may provide greater management value than retrospective estimation because future growth outcomes influence feeding, marketing, stocking, and grazing decisions [3]. Forecasting across multiple periods supports short-term operations and medium-term management planning [21].

Herd-based cattle live-weight (HB-CLW) forecasting predicts live weight for individual cattle within a herd. The framework uses observations collected under shared environmental and management conditions. The term “herd-based” refers to the production and data-collection context. The prediction target was the live weight of individual cattle rather than the average herd live weight. Published cattle live-weight forecasting studies have predominantly reported single-period forecasting performance or weight-estimation outcomes derived from controlled observational settings [20,22]. Limited evidence describes forecasting performance across multiple future periods using irregular automated weighing observations collected under commercial grazing conditions.

This study developed an ML framework for HB-CLW forecasting using irregular automated live-weight records from one commercial study herd. The framework compared temporal aggregation strategies for forecasting over one-, two-, and three-month periods. Climatic lag predictors represented delayed environmental associations with live weight.

Three primary contributions are presented. First, aggregation strategies were evaluated for HB-CLW forecasting using irregular automated observations. Second, forecasting performance was assessed over one-, two-, and three-month periods. Third, current-month, one-month-lag, and two-month-lag rainfall and temperature predictors were evaluated for forecasting individual live-weight progression. These variables were assessed as associative predictive signals within multivariable models, not as independent causal effects. Permutation importance and SHapley Additive exPlanations (SHAP) analyses supported biological interpretation of model outputs.

## 2. Materials and Methods

### 2.1. Study Design

This study used a longitudinal observational design based on automated live-weight records collected from grazing cattle at one commercial site. Experimental units consisted of individual cattle. Each observational unit represented one monthly aggregated record for an individual animal. Each record included the monthly aggregated live weight and the predictor variables used in model development. Forecasting outputs were generated for individual cattle managed within commercial grazing herds equipped with SOO weighing systems. Automated live-weight observations were recorded through voluntary platform access and aggregated monthly to reduce behavioural variation associated with irregular weighing frequency. All procedures complied with the standards approved by the Charles Sturt University (CSU) Animal Ethics Committee and with institutional livestock-monitoring guidelines. No experimental manipulation of animals, pasture allocation, or feeding regimes occurred during data collection.

### 2.2. Study Site and Animals

Cattle observations were conducted at the Global Digital Farm (GDF) of CSU, Australia. The study spanned three consecutive years and enabled evaluation of HB-CLW forecasting under varying seasonal and environmental conditions. Data collection formed part of routine farm operations and was approved by the CSU Animal Ethics Committee (Approval Numbers A21414 and A22404). The study included commercial beef cattle that had been weaned before data collection. Animals were not maintained as breeding stock during the monitoring period. Cattle were monitored from approximately six to fourteen months of age, representing the post-weaning growth phase commonly managed in commercial grazing systems. Table 1 summarises the study cohorts.

**Figure 1:**
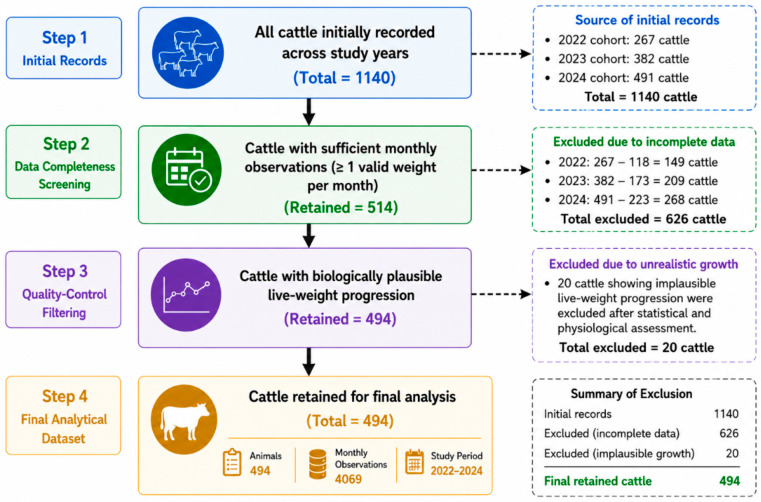
Sequential exclusion process for cattle records in the pre-processing pipeline.

Figure 1 summarises the sequential exclusion process applied during pre-processing. A total of 1140 cattle were initially recorded. Data completeness screening retained 514 cattle. Quality-control filtering excluded 20 cattle with biologically implausible live-weight trajectories. The final analytical dataset contained 494 cattle.

### 2.3. Data Sources

The three primary data sources comprised university livestock management records, automated in-paddock weighing data, and regional meteorological observations. University livestock management records provided electronic identification (EID), breed, sex, and date of birth. Individual live-weight measurements were collected using the Optiweigh system (Optiweigh Pty Ltd., Brisbane, QLD, Australia), which captures voluntary weight observations without physical restraint. Live-weight observations from conventional cattle-yard weighing sessions were not included. Regional rainfall and temperature observations were obtained from the Australian Bureau of Meteorology (BOM) website (The website of BOM (http://bom.gov.au, accessed on 2 August 2026)) to represent the environmental conditions affecting cattle growth [4].

Meteorological observations were obtained from the single BOM weather station located nearest the GDF study site. Monthly rainfall and temperature records were aggregated and temporally aligned with cattle live-weight observations before feature engineering and model development. Table 2 summarises all candidate variables, data sources, and modelling inclusion status.

### 2.4. Automated Weighing Infrastructure

**Figure 2:**
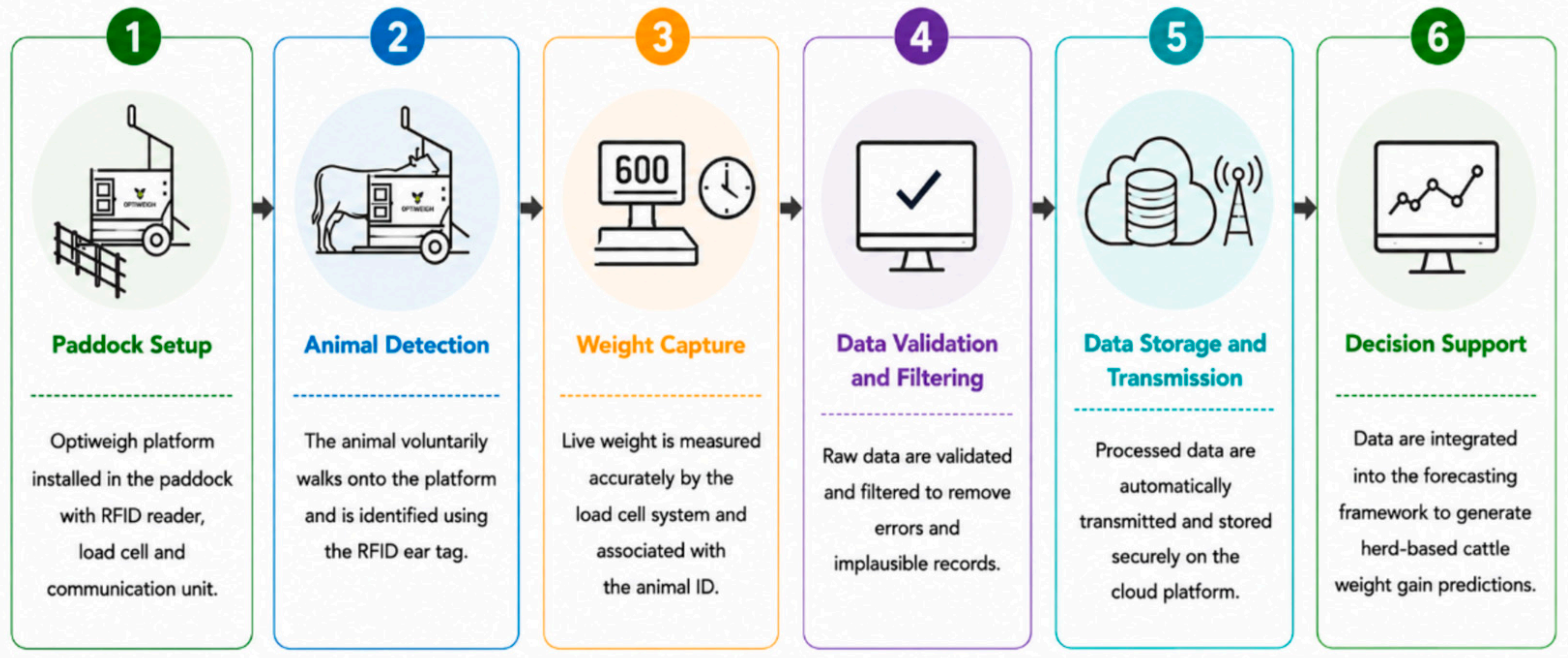
Operational workflow of the Optiweigh automated SOO weighing system used for cattle identification, live-weight acquisition, pre-processing, cloud synchronisation, and HB-CLW forecasting.

The Optiweigh system employs a front-leg weighing platform integrated with Radio-Frequency Identification (RFID) technology to identify individual cattle [8]. Cattle voluntarily place their front legs on a load-cell platform during normal movement. The system estimates whole-body live weight without physical restraint. Recorded measurements are automatically transferred to a remote online platform for continuous low-stress monitoring [10]. A total of 86,420 raw Optiweigh measurements were available before quality control and temporal aggregation. Figure 2 illustrates the Optiweigh workflow from cattle identification, live-weight acquisition, pre-processing, cloud synchronisation, and HB-CLW forecasting.

### 2.5. Data Preparation and Quality Control

The HB-CLW forecasting framework employed a pre-processing pipeline to ensure analytical consistency across all forecasting scenarios. Raw Optiweigh observations showed irregular sampling because cattle accessed the platform voluntarily [7]. Data completeness criteria were applied to ensure adequate temporal coverage. Each retained monthly period required at least one valid live-weight observation. Data completeness screening retained 514 cattle, including 118 from 2022, 173 from 2023, and 223 from 2024.

Point-level anomaly detection was applied to 86,420 raw Optiweigh measurements before temporal aggregation. Measurements were arranged chronologically for each animal. A rolling median absolute deviation procedure used a seven-observation window and a threshold of 3.5. Measurements exceeding this threshold were classified as point anomalies. The procedure removed 1296 measurements, representing 1.50% of all raw observations. Automated livestock weighing requires quality control because behavioural variability and platform noise can produce anomalous measurements [14].

Trajectory-level quality control was subsequently applied to the filtered measurements. Complete longitudinal trajectories were assessed for repeated changes inconsistent with expected biological growth. Persistent abnormalities indicated possible sensor malfunction, recording error, or unreliable platform visits [12]. The assessment excluded 20 cattle with biologically implausible trajectories.

After quality control, the final analytical dataset contained 494 cattle and 4069 monthly records. Only cattle meeting the predefined inclusion criteria were used for model development, validation, testing, and forecasting. Excluded cattle were omitted because their records did not satisfy the quality-control and longitudinal observation requirements. Monthly, weekly, and rolling-window datasets were reconstructed after point-level anomaly removal. Monthly aggregation then reduced behavioural noise and generated structured observations suitable for forecasting modelling [17].

Daily rainfall and temperature records were aggregated into monthly values and synchronised with live-weight records. A two-month climatic buffer was retained to construct lagged predictors [4]. Future live-weight observations were used only as target variables to minimise data leakage [23].

**Figure 3:**
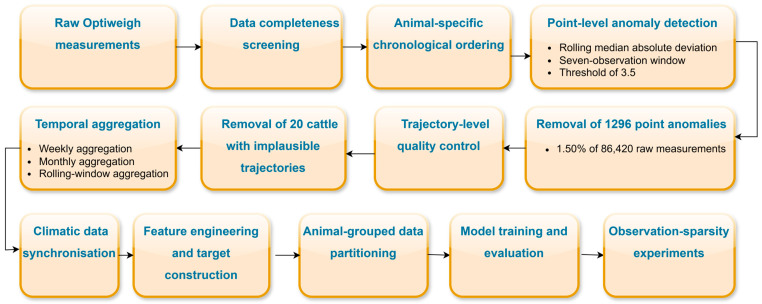
Data preparation workflow used to transform irregular automated livestock observations into structured monthly datasets for supervised ML forecasting under varying observation-sparsity conditions.

Forecasting robustness was assessed using observation-sparsity experiments. Each experiment removed 10% to 40% of observations across 30 iterations, retrained models, and preserved temporal order. Data augmentation was excluded because it may introduce unrealistic biological patterns into tabular livestock datasets [24]. All forecasting models and robustness experiments were rerun using datasets reconstructed after point-level anomaly removal. Figure 3 illustrates the pre-processing workflow used to transform irregular livestock observations into structured datasets for supervised ML analysis.

### 2.6. Temporal Aggregation Strategies

Three temporal aggregation strategies were implemented to transform irregular livestock observations into structured forecasting datasets. Weekly aggregation used fixed seven-day intervals. Monthly aggregation used calendar-month averages, whereas rolling-window aggregation used overlapping 30-day moving averages. The approaches standardised irregular observations before forecasting [16].

Information retention was evaluated using Shannon entropy calculated from aggregated live-weight distributions [25]. Entropy values were normalised relative to the original unaggregated observations. Variance preservation was assessed after aggregation. Forecasting robustness was evaluated under observation sparsity levels of 10%, 20%, 30%, and 40%.

Entropy retention was calculated using Equation (1).

(1) $$\begin{array}{c} H=-\sum_{i=1}^{n}p_{i}{log}_{2}(p_{i}) \end{array}$$

where *H* represents Shannon entropy, $p_{i}$ denotes the probability of observation class i, i represents the observation class index, and n represents the total number of observation classes. Entropy retention was expressed as the percentage of the aggregated data’s entropy relative to the original observations.

### 2.7. Feature Engineering and Target Construction

Feature engineering converted irregular observations into structured inputs for supervised ML models [26]. Candidate predictors formed predefined configurations for demographic, historical weight, and environmental comparisons. Dataset A included all evaluated predictors. Dataset B combined animal age with historical live weight. Dataset B served as the autoregressive baseline because it excluded weather variables. Dataset C added current and lagged rainfall and temperature to Dataset B. A comparison between Datasets B and C quantified the additional predictive contribution of weather information.

Animal age, expressed in months, was derived from birth date and monthly live-weight observations. Age was included in all dataset configurations because growth stage influences cattle development and live-weight progression [27].

Environmental predictors included monthly rainfall and temperature for the current month and two preceding months. Pearson correlations were used for exploratory screening before ML development [28]. The correlations did not test causality or establish valid time-series relationships. Models evaluated retained predictors simultaneously. Table 3 reports exploratory coefficients and nominal *p*-values. Final predictor inclusion depended on multivariable forecasting performance rather than correlation magnitude.

Rainfall exhibited the strongest positive association with cattle live weight at the one-month lag interval (r = 0.36, *p* < 0.001). Temperature exhibited moderate negative associations across all evaluated lag intervals. These exploratory results identified candidate climatic variables for subsequent machine learning evaluation rather than providing evidence of statistically robust time-series relationships. Correlation coefficients were interpreted only as preliminary indicators for feature selection. Final climatic predictors were selected according to multivariable forecasting performance rather than correlation magnitude. Climatic variables were incorporated as complementary predictors rather than primary determinants because historical live weight was expected to remain the dominant predictor of future live weight.

Multicollinearity among climatic predictors was evaluated using variance inflation factor analysis. Predictors with variance inflation factor values greater than 5 were excluded during model development [29].

Monthly aggregated live weight was calculated using Equation (2).

(2) $$W_{m}=\frac{1}{n}\sum_{i=1}^{n}w_{i}$$

where $W_{m}$ represents the monthly aggregated live weight for month m, $w_{i}$ denotes the *i*th valid live-weight observation recorded within month *m*, n represents the total number of valid observations recorded during month m, i represents the observation index, and m denotes the aggregation month.

Forecasting targets were generated using Equation (3).

(3) $$Y_{t+h}=W_{t+h}$$

where $Y_{t+h}$ represents the forecasting target at forecasting period h, $W_{t+h}$ denotes the future live-weight observation shifted forward by h months, t represents the current monthly observation, and h denotes the forecasting period (1, 2, or 3 months).

Lagged climatic predictors were generated using Equation (4).

(4) $$X_{climate}^{\left(t-k\right)}=\{Rainfall_{t-k},Temperature_{t-k}\},k\in \{0,1,2\}$$

where $X_{climate}^{\left(t-k\right)}$ represents a climatic predictor shifted by k monthly intervals, X denotes the climatic predictor, t represents the current month, and k denotes the lag interval. Lag intervals included the current month (k=0), previous first month (k=1), and previous second month (k=2).

Delayed climatic relationships were quantified using Equation (5).

(5) $$r_{k}=\frac{\sum_{t=1}^{n-k}(W_{t}-\bar{W})(C_{t-k}-\bar{C})}{\sqrt{\sum_{t=1}^{n-k}(W_{t}-\bar{W}{)}^{2}}\sqrt{\sum_{t=1}^{n-k}(C_{t-k}-\bar{C}{)}^{2}}}$$

where $r_{k}$ represents the cross-correlation coefficient at lag k, $W_{t}$ denotes the monthly cattle live weight at month t, $C_{t-k}$ represents the climatic observation shifted by lag interval k, t represents the current month, and k denotes the lag interval.

Three target variables were generated by forward-shifting the monthly aggregated live-weight observations to represent one-, two-, and three-month forecasting periods. The analytical dataset contained 4069 monthly records before target generation. Temporal shifting produced 3048 records for one-month forecasting, 2558 records for two-month forecasting, and 2068 records for three-month forecasting. Each forecasting period was evaluated independently using identical predictor configurations. Performance differences between Dataset B and Dataset C were used to quantify the additional predictive contribution of climatic variables beyond the baseline autoregressive model based on historical live weight.

### 2.8. Dataset Configuration

The processed dataset was divided into three experimental groups corresponding to one-month, two-month, and three-month forecasting periods. Five feature configurations, designated Dataset A through Dataset E, were defined for each forecasting period to evaluate the marginal contribution of demographic, historical live-weight, and environmental predictors. Table 4 summarises the predictor variables included within each dataset configuration. The experimental design enabled consistent comparison of predictor combinations across all forecasting periods.

### 2.9. ML Models

HB-CLW forecasting was evaluated using six supervised ML algorithms, including linear, tree-based, ensemble, kernel-based, and neural network approaches. The algorithms were Linear Regression (LR), Decision Tree (DT), Gradient Boosting (GB), Random Forest (RF), Neural Network (NN), and Support Vector Regression (SVR). Each model was evaluated across all forecasting periods and feature configurations. These methods are commonly used for predictive modelling in agricultural and livestock applications [30,31]. Predictive modelling and statistical analyses were implemented using Python version 3.12.4 (Python Software Foundation, Wilmington, DE, USA) and associated ML and statistical libraries.

Live weight target values were standardised using Z-score transformation before model training [32]. For each training subset generated during grouped cross-validation, the mean and standard deviation were calculated exclusively from the training data. The corresponding transformation parameters were then applied to the associated validation subset to prevent information leakage. Root Mean Square Error (RMSE) and Mean Absolute Error (MAE) were calculated after inverting the predicted and observed live weight values and are reported in kilograms for practical interpretation. Standardisation was applied only during model training and prediction. Model performance is reported using the original live weight scale.

### 2.10. Sequential Benchmark Models

Sequential benchmark models included Long Short-Term Memory (LSTM) and AutoRegressive Integrated Moving Average (ARIMA) models to provide a comparison with the proposed machine learning framework. The ARIMA model order (p, d, q) was selected using the minimum Akaike Information Criterion for each forecasting period. LSTM consisted of a single hidden layer with 64 units and ReLU activation, followed by a dense output layer. Model training used the Adam optimiser with a learning rate of 0.001, a batch size of 32, and 100 training epochs with early stopping based on validation loss. A one-month input sequence was used to predict each forecasting period. Both benchmark models were trained and evaluated using the same training, validation, and testing procedures as the proposed ML models to ensure fair performance comparison.

### 2.11. Model Validation and Statistical Analysis

Forecasting performance was evaluated using the coefficient of determination (R^2^), RMSE, and MAE. RMSE and MAE were calculated after inverting the predicted and observed live weight values and are reported in kilograms. R^2^ served as the principal scale-independent performance metric because it enables direct comparison among forecasting models. Model performance was evaluated separately for one-month, two-month, and three-month forecasting periods.

For each forecasting period, forecasting targets were created by forward temporal shifting of monthly live-weight observations before dataset partitioning. Predictor variables originated only from the current or preceding months. Future observations were used exclusively as response variables. Animal-grouped training and testing subsets were then created using animal identification as the grouping variable. This approach prevented records from the same animal from appearing in both subsets and reduced information leakage [33]. Grouped partitioning evaluated model generalisation across independent animals rather than chronological forecasting performance. Temporal train-test splitting was not implemented in the present study. Forward temporal shifting ensured that predictor variables were derived only from historical observations available at the time of prediction.

Hyperparameter optimisation was performed exclusively within the training subset using grouped 10-fold cross-validation. Standardisation parameters were estimated only from the training data within each cross-validation fold and subsequently applied to the corresponding validation data. Validation and test data were not used for feature scaling or model training. Following optimisation, the selected model was retrained using the complete training subset and evaluated on the independent test subset. Training and test performance metrics were calculated separately to assess model fit and generalisation. Prediction uncertainty was quantified using 95% prediction intervals derived from grouped cross-validation residuals [34]. Calibration error was calculated as the absolute difference between observed and expected interval coverage.

Residual diagnostics were conducted for GB using independent test predictions from each forecasting period. Residuals represented observed live weight minus predicted live weight. Residuals-versus-fitted plots were examined for systematic patterns and changes in error variance. Residual dispersion was compared below and above a fitted live weight of 450 kg. The Brown and Forsythe modification of Levene’s test was used to assess differences in variance between these groups. The test used the group median and a significance threshold of *p* < 0.05. Normalised median absolute deviation was also calculated for each group.

Cook’s distance was calculated for each LR model using its training observations. Values exceeding 4/*n* were classified as potentially influential. The term n represented the number of training observations within the corresponding forecasting dataset. Influential observations were linked to individual cattle using EID. This analysis quantified potentially influential records and the number of affected cattle.

Forecasting degradation was calculated relative to one-month forecasting performance using the following equation:

(6) $$\begin{array}{c} \mathrm{Forecasting}\;\mathrm{Degradation}\;(\%)=\frac{R_{1\text{-}\mathrm{month}}^{2}-R_{h}^{2}}{R_{1\text{-}\mathrm{month}}^{2}}\times 100 \end{array}$$

where $R_{1}^{2}$ represents the coefficient of determination $\left(R^{2}\right)$ for one-month forecasting, $R_{h}^{2}$ denotes the coefficient of determination $\left(R^{2}\right)$ for forecasting period h, and h represents the forecasting period corresponding to either two-month or three-month forecasting.

Comparative performance was assessed using mean cross-validation metrics. Forecasting robustness was evaluated through observation-sparsity experiments and comparison across forecasting periods [35].

### 2.12. Cross-Validation and Hyperparameter Optimisation

Grid search optimisation, integrated with grouped 10-fold cross-validation, was applied after dataset partitioning [36]. Hyperparameter selection prioritised maximum cross-validated R^2^ and minimum RMSE. LR was implemented using ordinary least squares estimation and required no hyperparameter optimisation.

Hyperparameter ranges were selected from previous ML studies and confirmed through preliminary experiments to capture stable model performance across all forecasting periods. Table 5 summarises the evaluated hyperparameter ranges and the final model configurations.

### 2.13. Explainability Analysis

Explainability analysis was conducted after final model selection and hyperparameter optimisation. Explainability methods were applied to the GB model because it consistently achieved the highest test R^2^ across the one-, two-, and three-month forecasting periods in Dataset A. RF achieved comparable performance. However, GB provided the highest overall predictive accuracy and more stable feature-importance rankings. Using a single model ensured consistent interpretation across forecasting periods.

Permutation importance analysis was first performed to rank predictor contributions [37]. Predictor importance was calculated by randomly shuffling each predictor’s values and measuring the resulting decrease in model performance. Larger reductions in predictive performance indicated greater predictor importance. Importance rankings were generated separately for one-month-, two-month-, and three-month-ahead forecasting experiments.

SHAP analysis was subsequently performed to evaluate the contribution of individual predictors to model predictions. SHAP values were calculated using the TreeSHAP algorithm implemented for tree-based ensemble models [38]. SHAP values quantified the direction and magnitude of each predictor’s contribution. Predictor contributions were examined across forecasting periods to assess ranking stability. Current-month and lagged rainfall and temperature variables were evaluated separately to quantify delayed climatic influences on cattle live-weight progression.

### 2.14. Automated Pre-Processing Platform

**Figure 4:**
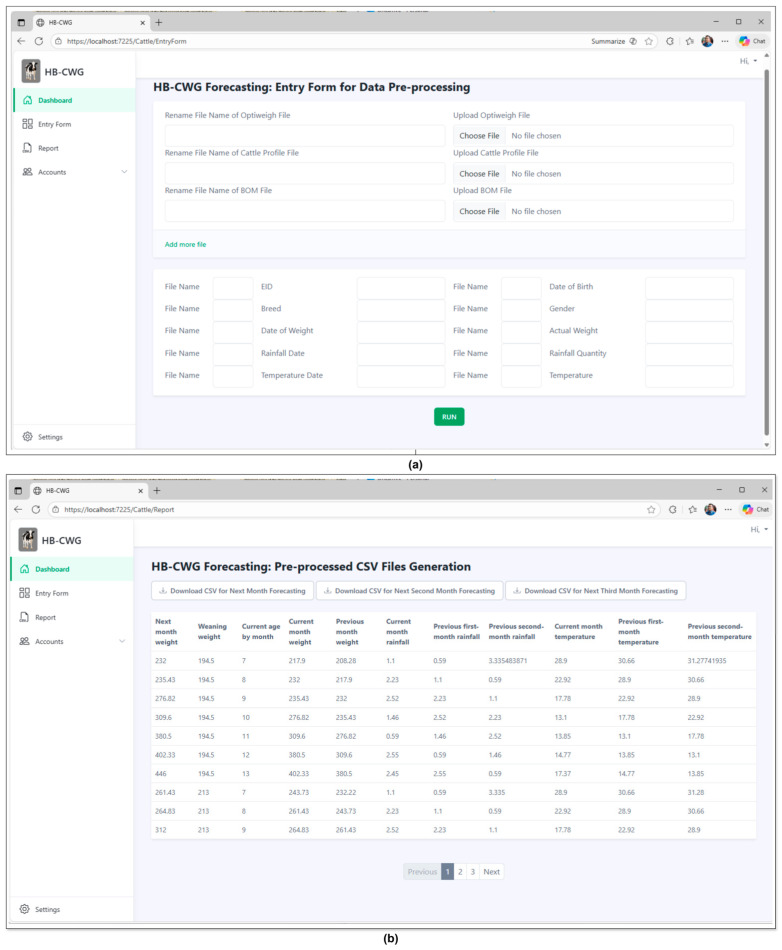
User interface of the automated pre-processing platform developed for dataset preparation and analytical workflow generation. (**a**) Data entry interface for uploading cattle live-weight and climatic data and initiating the automated pre-processing workflow. (**b**) Output interface showing the generated pre-processed dataset for forecasting analysis.

An automated pre-processing platform was developed to support reproducible implementation of the forecasting workflow. The platform performs data harmonisation, temporal alignment, and generates analytical datasets from livestock and climatic observations. The automated pre-processing platform (Version 1.0), developed in this study, is publicly available on GitHub (EXE file is available at https://github.com/Riaz-Hasib-Hossain/HB-CWG, accessed on 2 August 2026). Figure 4 presents the platform user interface.

## 3. Results

### 3.1. Dataset Characteristics and Aggregation Strategy Comparison

Point-level anomaly detection evaluated 86,420 raw Optiweigh measurements before temporal aggregation. The rolling median absolute deviation procedure identified 1296 anomalous measurements, representing 1.50% of the raw observations. These measurements were removed before constructing the weekly, monthly, and rolling-window datasets. Subsequent trajectory-level quality control excluded 20 cattle with persistent biologically implausible growth patterns.

The quality-control process retained 494 cattle and 4069 monthly records. The forecasting-period-specific datasets contained 3048, 2558, and 2068 records for one-month, two-month, and three-month forecasts, respectively. Monthly aggregation produced the strongest overall performance across entropy retention, variance preservation, and forecasting accuracy. Rolling-window aggregation ranked second, and weekly aggregation ranked lowest (Table 6).

Forecasting performance declined as observation sparsity increased. Monthly aggregation remained the most robust strategy across all observation-sparsity levels (Table 7).

**Figure 5:**
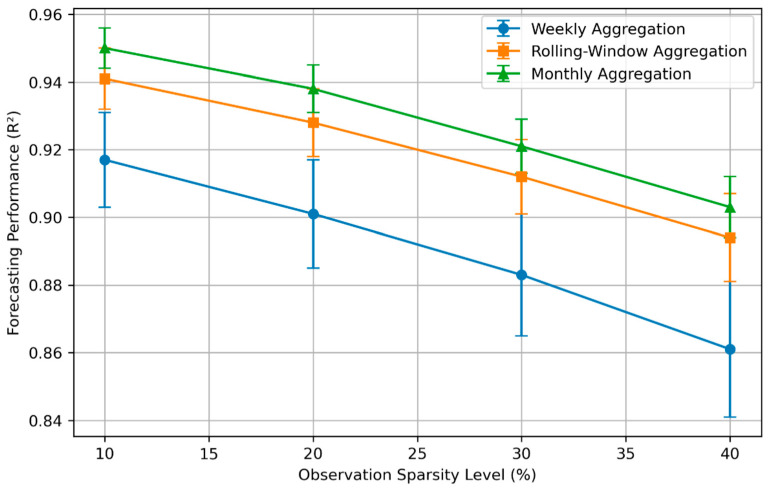
Forecasting performance across weekly, rolling window, and monthly aggregation strategies under increasing observation sparsity.

**Figure 6:**
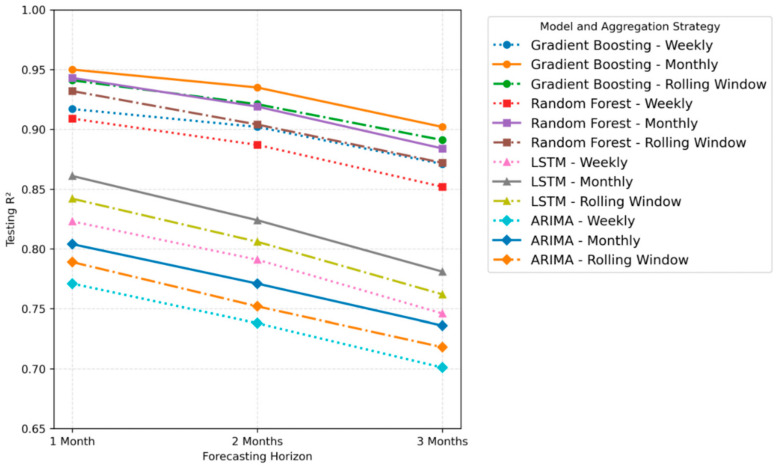
Testing R^2^ across aggregation strategies and sequential benchmarks for periods of one, two, and three months. Error bars show cross-validation standard deviations.

Figure 5 and Figure 6 illustrate the effects of observation sparsity and forecasting-period extension across aggregation strategies and sequential benchmarks.

### 3.2. Forecasting Performance Across One-Month, Two-Month, and Three-Month Forecasting Periods

Monthly aggregation produced the strongest information retention, accuracy, and robustness. Subsequent experiments used monthly records for one-, two-, and three-month periods. For the one-month forecasting period, GB achieved the highest one-month test performance. Its R^2^ was 0.950, RMSE was 3.82 kg, and MAE was 2.55 kg. RF ranked second. LSTM and ARIMA produced lower one-month accuracy. Table 8 summarises the comparative performance for the one-month forecasting period.

ML models consistently outperformed the sequential benchmark models across all forecasting periods. GB achieved the highest testing R^2^ values for Dataset A at the one-month, two-month, and three-month forecasting periods. RF produced the second-highest predictive performance and remained comparable with Gradient Boosting across several experiments. Prediction accuracy declined as the forecasting period increased. Ensemble methods maintained the strongest performance. Table 9, Table 10, Table 11 and Table 12 present the detailed training and testing results for each forecasting period and feature configuration.

### 3.3. Forecasting Stability Across Prediction Periods

Forecast uncertainty increased progressively as the forecasting period extended from 1 to 3 months. Mean prediction interval width increased from 3.26 kg for one-month forecasting to 4.31 kg for two-month forecasting and 5.08 kg for three-month forecasting. Calibration error increased from 0.018 to 0.039. Coverage of the 95% prediction intervals declined from 94.2% to 91.4%, indicating a gradual reduction in predictive certainty as the forecasting period increased.

GB achieved testing R^2^ values of 0.950, 0.935, and 0.902 across one-, two-, and three-month forecasting periods, respectively. RF achieved corresponding values of 0.943, 0.923, and 0.894. The high R^2^ values are consistent with the inclusion of recent live-weight predictors and the relatively stable within-farm data structure. These results represent internal forecasting performance and should not be interpreted as external validation.

Dataset configurations incorporating climatic variables often improved ensemble-based forecasts, particularly at two- and three-month periods. These improvements varied across model architectures and predictor compositions. Climatic variables should be interpreted as complementary predictive factors, rather than as universally beneficial predictors. Forecast uncertainty and calibration metrics are summarised in Table 13.

**Figure 7:**
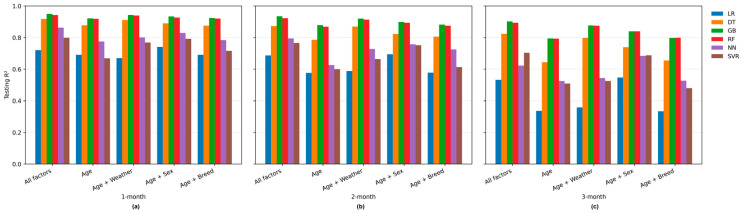
Testing R^2^ values of ML models across one-, two-, and three-month forecasting periods.

**Figure 8:**
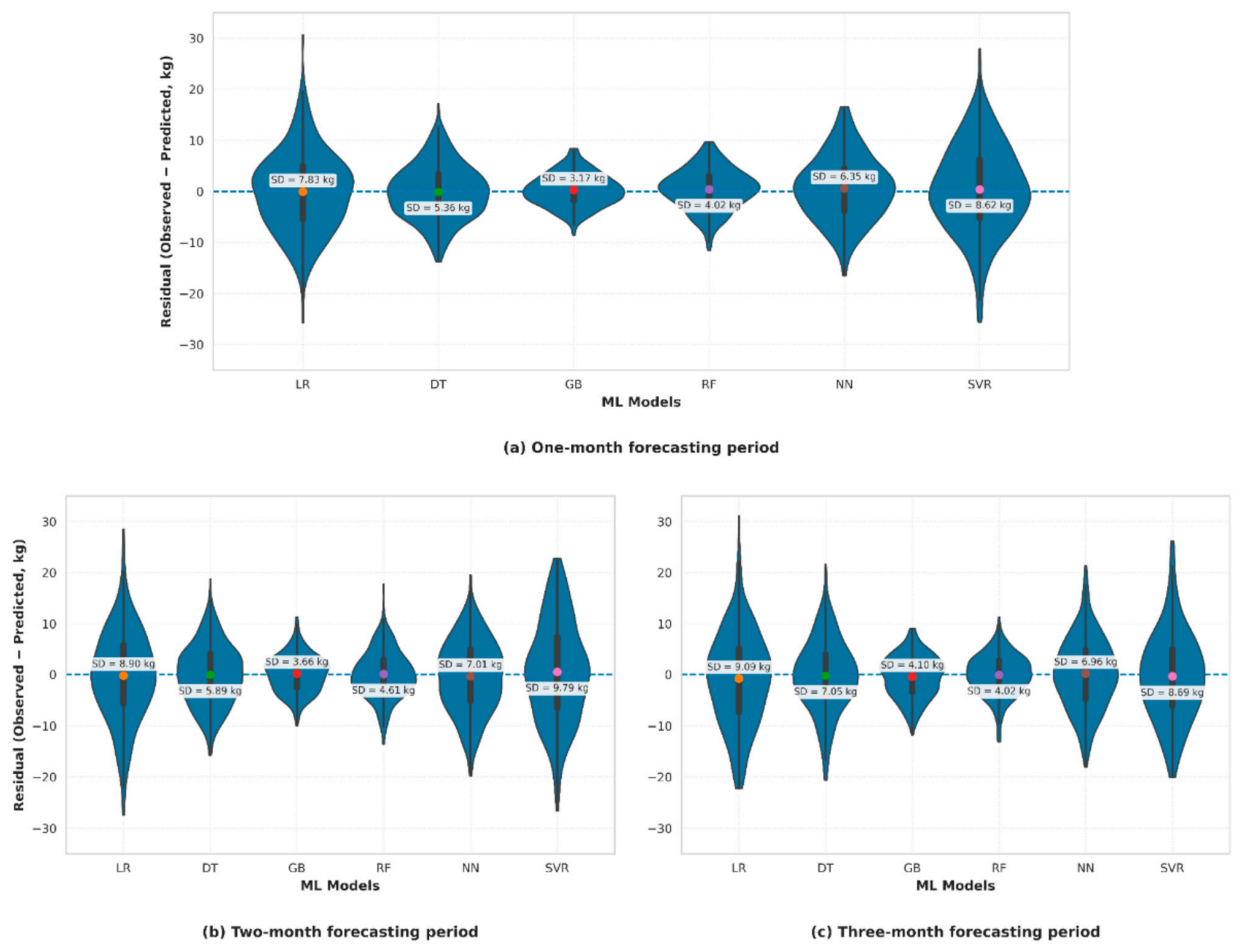
Distribution of prediction residuals for the evaluated machine learning models across one-month, two-month, and three-month forecasting periods. Residuals are reported in kilograms following inverse transformation of the standardised prediction errors. Wider distributions indicate greater prediction uncertainty.

**Figure 9:**
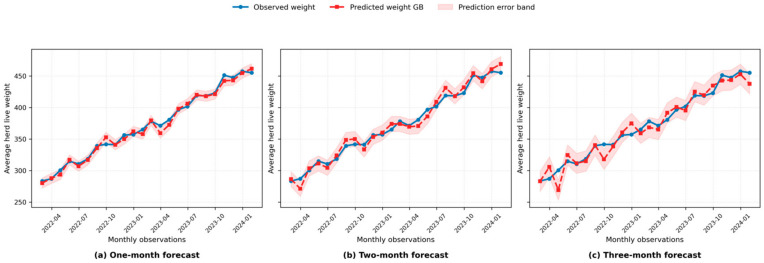
Monthly herd mean observed and GB-predicted live weights across the displayed evaluation period. The shaded band represents the original model uncertainty output.

**Figure 10:**
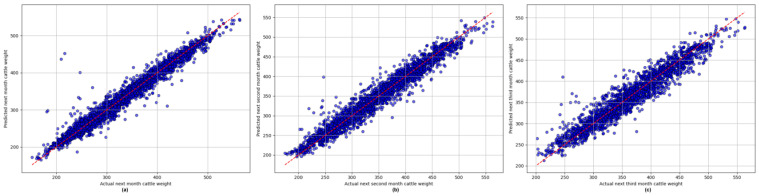
Observed versus predicted live weights generated using the GB model across forecasting periods.

Figure 7, Figure 8, Figure 9 and Figure 10 provide visual comparisons of testing accuracy, forecasting-error distributions, temporal prediction behaviour, and observed-versus-predicted live-weight relationships across forecasting periods. Predicted values remained closely aligned with observed live weights, although prediction dispersion increased moderately at longer forecasting intervals.

### 3.4. Feature-Importance Analysis

Feature-importance analysis identified age and previous live weight as the dominant predictors across all forecasting periods (Table 14). Age contributed importance scores of 0.41, 0.39, and 0.37 under one-, two-, and three-month forecasting, respectively, whereas previous live weight contributed importance scores of 0.33, 0.31, and 0.29. These predictors provided the strongest information for forecasting future live weight.

Environmental importance increased as forecasting periods lengthened. The importance of combined rainfall increased from 0.11 to 0.16, while the contribution of combined temperature also increased, from 0.09 to 0.13. The first-month rainfall lag remained the strongest individual rainfall predictor, with its SHAP value increasing from 0.055 to 0.113. The importance of temperature lag variables also increased across forecasting periods. In contrast, sex and breed contributed little to model predictions.

SHAP analysis further demonstrated increasing contributions from lagged climatic variables under medium-term forecasting. The first-month rainfall had the greatest climatic importance in three-month forecasting, followed by the previous second-month rainfall. Similar patterns were observed for lagged temperature variables. Lagged climatic predictors became increasingly informative as forecasting periods extended. Table 15 summarises the SHAP importance values for climatic lag predictors.

**Figure 11:**
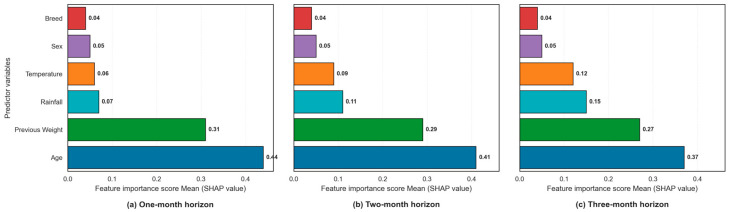
Feature-importance visualisation generated using the GB model. Climatic predictors are displayed separately by current-month, one-month lag, and two-month lag intervals to maintain consistency with the feature-engineering procedure and SHAP analysis.

The combined feature-importance and SHAP analyses indicate that historical live-weight information, age, and lagged climatic conditions collectively underpinned medium-term forecasting performance in grazing cattle systems. Figure 11 presents a visual summary of predictor contributions derived from the GB model.

### 3.5. Residual and Influence Diagnostics

Residuals-versus-fitted plots showed that GB residuals remained centred near zero across all forecasting periods. Mean residuals were 0.08 kg, 0.14 kg, and 0.21 kg for the respective forecasting periods. No strong systematic curvature was evident. However, residual dispersion increased among fitted live weights of at least 450 kg.

For one-month forecasting, normalised median absolute deviation increased from 2.85 kg below 450 kg to 5.62 kg at higher fitted weights. The Brown and Forsythe test identified unequal residual variances at the 450 kg threshold (F = 14.73, *p* < 0.001). For two-month forecasting, the corresponding dispersion values were 3.11 kg and 6.24 kg. Residual variances also differed significantly for this forecasting period (F = 16.89, *p* < 0.001). For three-month forecasting, dispersion increased from 3.38 kg to 7.02 kg. The variance difference remained significant (F = 19.41, *p* < 0.001). These findings indicate greater forecasting uncertainty among heavier cattle.

Cook’s distance analysis identified 37 potentially influential LR observations for one-month forecasting. These observations represented approximately 1.5% of the training data and originated from 24 cattle. The two-month analysis identified 34 influential observations, representing approximately 1.7%, from 22 cattle. The three-month analysis identified 31 influential observations, representing approximately 1.9%, from 20 cattle. Maximum Cook’s distance values were 0.021, 0.027, and 0.034, respectively. Influential records were distributed across multiple cattle rather than concentrated within one animal. Table 16 summarises the residual and influence diagnostics across the one-, two-, and three-month forecasting periods.

**Figure 12:**
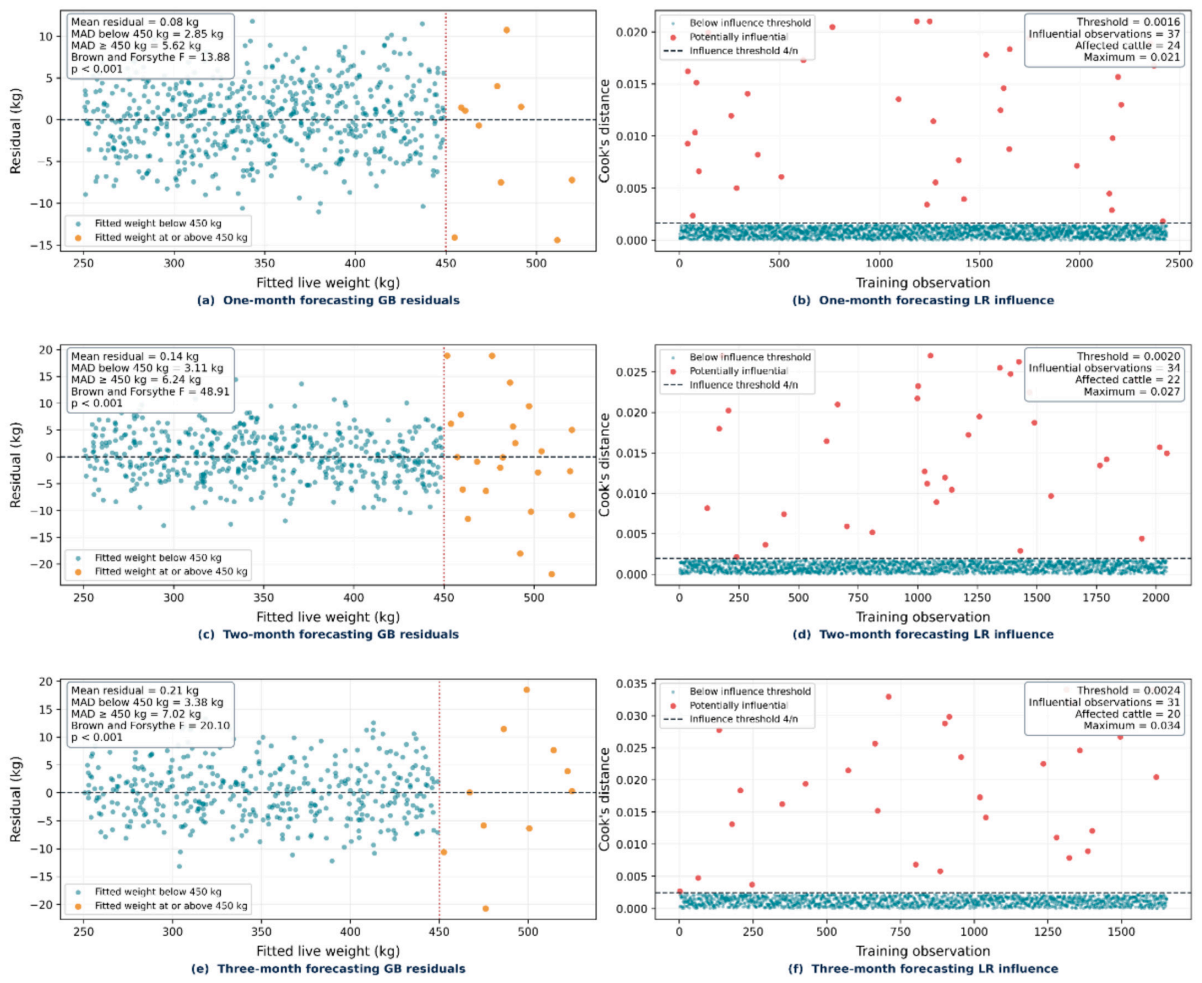
Residual dispersion and influential-observation diagnostics across the one-, two-, and three-month forecasting periods. The left panels show GB residuals against fitted live weight, and the right panels show LR Cook’s distance values.

In Figure 12, the panels show GB residuals against fitted live weights and LR Cook’s distance values. Horizontal reference lines indicate zero residuals and the corresponding 4/n influence thresholds. A vertical reference line identifies the fitted live-weight threshold of 450 kg.

## 4. Discussion

### 4.1. Principal Findings

The present study developed an ML framework for HB-CLW forecasting across multiple forecasting periods using automated observations collected from herd-managed grazing systems. Forecasts were generated for individual cattle managed within a study herd. Forecasting performance was evaluated across one-month, two-month, and three-month forecasting periods using demographic, historical live-weight, and climatic predictors. GB consistently achieved the strongest predictive performance across all forecasting periods. Monthly aggregation produced higher forecasting accuracy than weekly and rolling-window aggregation strategies. Climatic lag variables contributed increasingly to forecasting performance as forecasting periods extended. These findings demonstrate the value of automated livestock monitoring and ML for medium-term HB-CLW forecasting under commercial grazing conditions.

### 4.2. Aggregation Strategy Performance Under Commercial Grazing Conditions

Monthly aggregation consistently outperformed weekly and rolling-window strategies. It retained more entropy and variance and reduced missing-data propagation. Automated weighing produces irregular observations because cattle visit the platform voluntarily [17]. Short-term live weight variation reflects rumen fill, gastrointestinal contents, water intake, feeding behaviour, and hydration status. Monthly aggregation reduces these transient effects and better represents underlying growth. Weekly aggregation retains more short-term variation and reduces forecasting stability. Monthly aggregation also remained more stable as observation sparsity increased. Comparable advantages have been reported for automated livestock systems with irregular sampling [16].

### 4.3. Comparison with Previous Forecasting Studies

Ensemble-based ML approaches consistently outperformed linear, neural network, kernel-based, and sequential forecasting approaches across all forecasting periods. GB achieved testing R^2^ values of 0.950, 0.935, and 0.902 for the one-, two-, and three-month forecasting periods, respectively. RF produced comparable forecasting performance. Several previous studies have reported strong predictive performance of ensemble-learning approaches in agricultural forecasting because these methods effectively capture non-linear relationships among variables [19]. The present findings support these observations and demonstrate the suitability of ensemble-based methods for forecasting cattle live weight under commercial grazing conditions.

LR and SVR produced lower accuracy than the tree ensembles. LR could not represent non-linear growth relationships through its linear form. SVR used a Radial Basis Function (RBF) kernel, but its selected configuration captured less variation than those of GB and RF. Linear regression served only as a predictive benchmark. GB residuals remained centred near zero and showed no strong systematic curvature. However, residual variance increased significantly above a fitted live weight of 450 kg across all forecasting periods. Normalised median absolute deviation was approximately twice as large among heavier cattle. Forecasts for heavier cattle should consequently be interpreted with greater caution.

Cook’s distance analysis identified potentially influential observations across the three LR models. These records represented less than 2% of each training dataset and were distributed across multiple cattle. The results suggest that isolated observations influenced the LR benchmark. However, no single animal consistently dominated the regression results across all forecasting periods. The lower LR performance primarily reflected its limited representation of non-linear cattle growth relationships.

Sequential benchmarks produced lower predictive performance than structured ML approaches [39]. LSTM and ARIMA also declined more rapidly as forecasting periods increased. Commercial grazing systems often contain missing observations and irregular sampling intervals [40]. Sequence-dependent approaches perform best with continuous observations. Structured feature engineering and temporal aggregation improve robustness when observations are irregular.

Dataset B served as the autoregressive baseline because it included age and historical live weight, without weather predictors. Dataset C added current and lagged weather variables. Historical live weight remained the dominant predictor because cattle growth changes gradually.

For one-month forecasts, Dataset C reduced the GB test RMSE from 5.01 kg to 4.14 kg. MAE declined from 3.58 kg to 2.78 kg. The reductions were 0.87 kg and 0.80 kg, respectively (Table 9). For two-month forecasts, RMSE declined from 5.52 kg to 4.39 kg. MAE declined from 4.16 kg to 3.33 kg. The reductions were 1.13 kg and 0.83 kg, respectively (Table 11). For three-month forecasts, RMSE declined from 5.77 kg to 4.33 kg. MAE declined from 4.33 kg to 3.30 kg. The reductions were 1.44 kg and 1.03 kg, respectively (Table 12).

These improvements in prediction accuracy were modest because historical live weight captured most of the variation. Their practical value depends on the availability of weather data, herd size, and management objectives. A baseline model remains suitable when local weather data are unavailable. Weather predictors may provide additional value for farms that already collect meteorological data. A cost–benefit analysis should determine whether the observed reductions in prediction error justify the additional data integration.

Forecasting performance gradually declined as the forecasting period increased from 1 to 3 months. Such reductions are expected because biological and environmental uncertainty increases with the length of the prediction interval [6]. Despite this decline, forecasting accuracy remained high across all forecasting periods. Ensemble-learning approaches maintained strong medium-term forecasting performance under commercial grazing conditions.

Machine learning models were trained using Z-score standardisation, although all reported RMSE and MAE values were inverse-transformed and presented in kilograms to improve practical interpretation. Reporting prediction errors in the original measurement scale facilitates interpretation by producers, veterinarians, and livestock researchers. The coefficient of determination remained an important complementary performance measure because it enables comparison of predictive accuracy across different forecasting experiments [41].

### 4.4. Biological Interpretation of Climatic Lag Effects

The relative importance assigned to climatic variables increased for two- and three-month forecasts compared with one-month forecasts. SHAP analysis further identified the increasing relative importance of lagged climatic predictors across extended forecasting intervals. Climatic predictors should be interpreted within the forecasting framework, not as evidence of independent effects on cattle growth. Because predictor configurations differed, climatic relevance was inferred primarily from permutation importance and SHAP analyses in the final multivariable models. Historical live weight remained the dominant predictor, although climatic variables provided complementary information for medium-term forecasting.

Rainfall influences pasture growth and quality [39]. Temperature influences pasture productivity, forage quality, grazing behaviour, and metabolic demand. These processes can operate over several weeks. Lagged climate information may support forecasts of future live weight [18]. The explainability results indicate complementary predictive value rather than causal effects. Future studies should test these associations using external datasets, pasture measurements, and controlled comparisons of predictors. Kilogram-scale error reductions should be validated across farms.

### 4.5. Herd-Management Implications

The proposed forecasting framework has several practical applications for commercial grazing enterprises. Forecast-guided live-weight estimation can support feed allocation during changing pasture availability [42]. Earlier identification of reduced growth trajectories can facilitate timely nutritional intervention before substantial production losses occur [21].

Forecasting over multiple periods can also support pasture management planning [42]. Forecasted live-weight trajectories provide information on expected animal growth under prevailing environmental conditions and can assist grazing rotation and pasture utilisation decisions.

Supplementation programs may benefit from forecasting outputs because predicted reductions in live-weight progression can indicate when additional nutritional support is required [43]. Earlier intervention may improve feed-use efficiency and reduce seasonal growth setbacks.

Forecasting information may also support livestock marketing decisions by improving sale scheduling, transport planning, and market readiness assessments [21]. Similar decision-support opportunities have been reported in precision livestock farming research [43].

### 4.6. Study Limitations and Future Research

Several sample and methodological limitations should be considered when interpreting the findings. The proposed forecasting framework was developed and evaluated using data from a single commercial grazing enterprise, which may limit its applicability to other production environments, breeds, and management systems. Independent external validation using multi-farm datasets is required to confirm model robustness and generalisability. Climatic information was obtained from a single BOM weather station located near the study site. Local environmental variation and management factors were not incorporated into the forecasting framework. The irregular nature of automated weighing records also reduced the number of observations available for model development because animals with insufficient longitudinal records or biologically implausible growth trajectories were excluded during quality control.

The final analytical dataset retained 494 of 1140 recorded cattle, representing 43.3% of the initial cohort. The retention rate may favour animals that regularly visit the weighing platform. Such a selection may limit application to cattle with sparse voluntary observations. Greater eligibility may be achieved through improved platform placement, animal acclimation, attractant management, and routine equipment calibration. Longer monitoring periods and automated quality alerts may also improve longitudinal completeness and enable timely correction of measurement failures. Future studies should evaluate these strategies and report their effects on cattle inclusion rates and forecasting generalisability. Upper live weights shown in Figure 10 require interpretation by age, breed, sex, and castration status. Future validation should compare retained and excluded cattle across these characteristics.

The present study evaluated forecasting performance across the complete analytical dataset rather than separate animal growth stages. Model accuracy during early and later growth phases was not investigated because the primary objective was to develop a general herd-based forecasting framework. The growth stage may influence prediction accuracy because biological growth patterns change as animals mature. Future research should evaluate age-specific forecasting performance across early, middle, and later growth stages.

Residual diagnostics identified greater prediction variability among cattle with fitted live weights of at least 450 kg. Consequently, overall performance may not represent equivalent forecasting reliability across the complete live-weight range. The limited number of heavier cattle may have contributed to this increased uncertainty. External validation should include more heavier cattle across different breeds, sexes, and management conditions.

Future research should evaluate the proposed framework using independent datasets collected from multiple farms, breeds, production systems, and climatic regions. Additional environmental, nutritional, pasture, and management variables may further improve forecasting performance. Advanced deep-learning architectures and hybrid forecasting approaches should also be investigated to improve predictive accuracy and model generalisability. These investigations will strengthen confidence in practical implementation across diverse production environments.

## 5. Conclusions

This study developed and evaluated an ML framework for forecasting across multiple forecasting periods using irregular automated weighing observations collected under commercial grazing conditions. Individual cattle’s live weight was forecast using demographic attributes, historical live-weight records, and climatic predictors derived from cattle managed within the study herd.

GB achieved the highest forecasting performance across the one-, two-, and three-month forecasting periods. Monthly aggregation outperformed weekly and rolling-window aggregation by preserving more information and maintaining greater robustness under observation sparsity. Animal age and historical live weight were the most influential predictors. Lagged rainfall and temperature variables provided complementary predictive information, particularly within ensemble-based models and longer forecasting periods. Their contribution remained smaller than that of age and historical live-weight predictors, which formed the baseline autoregressive forecasting model. Climatic variables improved forecasting robustness under commercial grazing conditions. The climatic contribution should be interpreted as associative predictive information rather than evidence of direct climatic effects on cattle growth.

The study extends precision livestock forecasting research by evaluating individual-animal live-weight prediction within herd-managed grazing systems using irregular observations collected through automated weighing infrastructure. The findings demonstrate strong internal forecasting performance for individual cattle managed within the study herd under the evaluated commercial grazing conditions. Generalisation of the proposed framework to other farms, breeds, production systems, climatic regions, or geographical locations requires independent external validation using additional datasets.

The proposed framework provides a practical approach for integrating automated livestock monitoring, climatic information, and ML into precision livestock farming systems. Forecasting outputs may support proactive grazing management, nutritional planning, pasture allocation, and early identification of growth deviations. Broader adoption of ML-based forecasting frameworks may improve resource utilisation, animal welfare, production sustainability, and evidence-based decision-making under variable grazing conditions. Future research should evaluate external model performance across diverse production environments to confirm the robustness and generalisability of the proposed framework.

## Figures and Tables

**Table 1:** Characteristics of cattle cohorts included in the study across observation years.

Year	Number of Animals (n)	Breed Composition	Sex Distribution	Birth Period	Data Collection Duration
2022	267	Angus	Male	July 2021 to September 2021	February 2022 to October 2022
2023	382	Angus and Limousin	Male and Female	July 2022 to October 2022	February 2023 to September 2023
2024	491	Angus and Limousin	Male and Female	July 2023 to September 2023	March 2024 to October 2024

**Table 2:** Candidate variables used in HB-CLW forecasting model development.

Variable	Category	Source	Included in Model Development	Justification
EID	Identifier	University records	No	Used for record alignment and animal identification
Breed	Demographic	University records	Yes	Captures genetic variation among animals
Sex	Demographic	University records	Yes	Represents biological growth variation
Date of Birth	Demographic	University records	Yes	Used to calculate animal age
Rainfall	Environmental	BOM	Yes	Influences pasture growth and feed availability
Temperature	Environmental	BOM	Yes	Influences metabolic response and growth performance
Temperature Date	Temporal Alignment	BOM	No	Used for temporal synchronisation before monthly aggregation
Rainfall Date	Temporal Alignment	BOM	No	Used for temporal synchronisation before monthly aggregation
Weigh Date	Temporal Alignment	Optiweigh	No	Used for temporal aggregation and record alignment
Live Weight	Response Variable	Optiweigh	Yes	Target variable for forecasting model development

**Table 3:** Exploratory Pearson correlations between monthly live weight and current or lagged climatic variables.

Variable	Current-Month r	Current-Month *p*-Value	Lag 1 Month r	Lag 1 Month *p*-Value	Lag 2 Months r	Lag 2 Months *p*-Value
Rainfall	0.21	<0.05	0.36	<0.001	0.31	<0.01
Temperature	−0.14	<0.05	−0.22	<0.01	−0.19	<0.05

Note: Coefficients and nominal *p*-values came from unadjusted two-tailed tests. Serial dependence and repeated animal observations limit inferential interpretation.

**Table 4:** Feature configurations used for HB-CLW forecasting across multiple forecasting periods.

Feature Group	Variable	Dataset A: All Factors	Dataset B: Age + Historical Weight	Dataset C: Age + Weather + Historical Weight	Dataset D: Age + Sex + Historical Weight	Dataset E: Age + Breed + Historical Weight
Demographic	Age	✓	✓	✓	✓	✓
	Sex	✓	✗	✗	✓	✗
	Breed	✓	✗	✗	✗	✓
Historical weight	Previous-Month Weight	✓	✓	✓	✓	✓
	Current-Month Weight	✓	✓	✓	✓	✓
Environmental	Rainfall (Current Month)	✓	✗	✓	✗	✗
	Rainfall (Previous First-Month)	✓	✗	✓	✗	✗
	Rainfall (Previous Second-Month)	✓	✗	✓	✗	✗
	Temperature (Current Month)	✓	✗	✓	✗	✗
	Temperature (Previous First-Month)	✓	✗	✓	✗	✗
	Temperature (Previous Second-Month)	✓	✗	✓	✗	✗

Note: Age was expressed in months. Previous-month weight represents the live weight recorded during the preceding month. Current-month weight represents the live weight recorded during the current aggregation period. The previous first-month and the previous second-month represent climatic observations lagged by 1 month and 2 months, respectively. Dataset configurations were designed to evaluate practical predictor combinations for forecasting rather than to isolate the independent effects of specific predictor groups. ✓ indicates that the variable was included in the corresponding dataset configuration. ✗ indicates that the variable was not included.

**Table 5:** Hyperparameter optimisation ranges, optimisation strategy, and selected final configurations for supervised ML models used in HB-CLW forecasting.

Model	Evaluated Hyperparameters	Search Range	Final Model Configuration
LR	None	Not applicable	Ordinary Least Squares
DT	Maximum depth, minimum samples split, criterion	Depth = 2–20	max_depth = 10, min_samples_split = 4
GB	Number of estimators, learning rate, and tree depth	Estimators = 50–500	n_estimators = 300, learning_rate = 0.05, max_depth = 4
RF	Number of estimators, maximum depth	Estimators = 50–500	n_estimators = 250, max_depth = 12
NN	Hidden layers, neurons, activation function, learning rate, batch size, dropout rate, epochs	Layers = 1–3; Neurons = 32–256; Learning rate = 0.0001–0.01; Batch size = 16–128; Dropout = 0.10–0.50; Epochs = 50–300	hidden_layer_sizes = (100,50), activation = relu, learning_rate = 0.001, batch_size = 32, dropout = 0.20, epochs = 150
SVR	C parameter, gamma, kernel	C = 0.001–100	C = 10, gamma = 0.01, kernel = rbf

**Table 6:** Comparative methodological evaluation of aggregation strategies using entropy retention, variance preservation, missing-data propagation, and forecasting accuracy under irregular automated livestock observations.

Aggregation Strategy	Entropy Retention (%)	Variance Preservation (%)	Missing-Data Propagation (%)	Mean R^2^
Weekly	71.4	68.2	34.8	0.917
Monthly	89.6	91.3	12.6	0.950
Rolling Window	84.2	86.7	18.9	0.941

Note: The calculation procedures for entropy retention, variance preservation, and missing-data propagation are described in the Section 2.

**Table 7:** Forecasting degradation under repeated random observation sparsity across aggregation strategies.

Observation Sparsity Level	Weekly Aggregation R^2^	Rolling-Window Aggregation R^2^	Monthly Aggregation R^2^
10%	0.902	0.929	0.941
20%	0.884	0.913	0.928
30%	0.861	0.894	0.909
40%	0.833	0.871	0.887

Note: Values represent forecasting performance after random observation removal and model retraining at each sparsity level.

**Table 8:** Comparative one-month forecasting performance across aggregation strategies and sequential benchmarks.

Forecasting Method	Aggregation Strategy	Period	Number of Records	R^2^ (Mean ± SD)	RMSE (kg) (Mean ± SD)	MAE (kg) (Mean ± SD)
GB	Monthly	1 Month	3048	0.950 ± 0.006	3.82 ± 0.32	2.55 ± 0.24
GB	Weekly	1 Month	3048	0.917 ± 0.014	5.49 ± 0.56	4.06 ± 0.40
GB	Rolling Window	1 Month	3048	0.941 ± 0.009	4.30 ± 0.40	3.10 ± 0.32
LSTM	Sequential	1 Month	3048	0.861 ± 0.017	7.48 ± 0.64	5.33 ± 0.48
ARIMA	Sequential	1 Month	3048	0.804 ± 0.022	9.31 ± 0.88	6.61 ± 0.64

Note: RMSE and MAE are reported in kilograms following inverse transformation of the standardised prediction errors to improve the practical interpretation of forecasting performance. Higher R^2^ values indicate better predictive performance. Lower RMSE and MAE values indicate smaller prediction errors.

**Table 9:** Accuracy assessment of one-month-ahead HB-CLW forecasting using training and testing datasets.

Dataset Configuration	Model	R^2^ Training	RMSE Training (kg)	MAE Training (kg)	R^2^ Testing	RMSE Testing (kg)	MAE Testing (kg)
Dataset A: All Factors	LR	0.872	6.68	4.62	0.720	8.35	4.77
	DT	0.921	4.69	2.47	0.918	5.09	3.26
	GB	0.963	3.18	2.31	0.950	3.82	2.55
	RF	0.946	3.66	2.47	0.943	4.06	2.63
	NN	0.868	7.08	3.02	0.863	5.57	3.26
	SVR	0.802	9.39	8.28	0.799	8.43	6.68
Dataset B: Age + Historical Weight	LR	0.813	8.12	5.25	0.691	8.91	5.33
	DT	0.881	6.05	4.38	0.878	6.45	4.46
	GB	0.944	4.22	3.18	0.922	5.01	3.58
	RF	0.921	4.62	3.34	0.919	5.01	3.50
	NN	0.778	7.48	4.85	0.775	7.88	4.93
	SVR	0.673	10.82	8.59	0.669	11.06	8.83
Dataset C: Age + Weather + Historical Weight	LR	0.835	7.64	4.85	0.670	9.07	5.01
	DT	0.914	4.93	3.34	0.911	5.33	3.42
	GB	0.959	3.42	2.47	0.943	4.14	2.78
	RF	0.943	3.82	2.63	0.940	4.22	2.71
	NN	0.806	6.45	3.74	0.801	7.00	3.98
	SVR	0.772	8.91	7.00	0.769	9.07	7.16
Dataset D: Age + Sex + Historical Weight	LR	0.855	7.16	4.93	0.741	8.28	5.09
	DT	0.893	5.65	3.26	0.890	6.05	4.14
	GB	0.949	3.90	3.02	0.934	4.54	3.34
	RF	0.931	4.38	3.10	0.927	4.77	3.26
	NN	0.832	6.68	4.38	0.829	7.08	4.54
	SVR	0.795	8.35	6.60	0.792	8.59	6.76
Dataset E: Age + Breed + Historical Weight	LR	0.814	8.12	5.25	0.691	8.91	5.33
	DT	0.879	6.05	4.38	0.876	6.52	4.46
	GB	0.945	4.14	3.10	0.924	4.93	3.50
	RF	0.924	4.62	3.26	0.921	5.01	3.42
	NN	0.770	6.84	4.22	0.784	7.40	4.54
	SVR	0.719	9.95	7.88	0.716	10.18	8.04

Note: RMSE and MAE are reported in kilograms following inverse transformation of the standardised prediction errors to improve the practical interpretation of forecasting performance. Higher R^2^ values indicate better predictive performance. Lower RMSE and MAE values indicate smaller prediction errors.

**Table 10:** Forecasting performance under progressive observation sparsity for ML and sequential forecasting models.

Missing Observation Level	GB R^2^	LSTM R^2^	ARIMA R^2^
10%	0.947	0.852	0.798
20%	0.938	0.821	0.762
30%	0.921	0.783	0.724
40%	0.894	0.741	0.691

Note: Higher R^2^ values indicate better forecasting performance. Increasing observation sparsity reduced predictive accuracy across all forecasting approaches. Gradient Boosting maintained the highest predictive robustness under all sparsity levels.

**Table 11:** Accuracy assessment of two-month-ahead HB-CLW forecasting using training and testing datasets.

Dataset Configuration	Model	R^2^ Training	RMSE Training (kg)	MAE Training (kg)	R^2^ Testing	RMSE Testing (kg)	MAE Testing (kg)
Dataset A: All factors	LR	0.834	6.58	4.77	0.687	8.02	4.84
	DT	0.877	5.22	3.93	0.873	5.60	4.01
	GB	0.947	3.48	2.65	0.935	3.86	2.95
	RF	0.927	4.31	2.95	0.923	4.24	3.03
	NN	0.800	5.45	3.33	0.795	6.05	3.56
	SVR	0.770	7.72	5.98	0.766	7.94	6.28
Dataset B: Age + historical weight	LR	0.719	8.70	5.98	0.576	9.46	6.05
	DT	0.816	7.11	5.14	0.786	7.49	5.22
	GB	0.906	4.84	3.63	0.879	5.52	4.16
	RF	0.873	5.30	4.01	0.869	5.75	4.16
	NN	0.629	8.62	5.52	0.626	9.00	5.67
	SVR	0.630	10.29	8.40	0.600	10.44	8.55
Dataset C: Age + weather + historical weigh	LR	0.772	7.79	5.30	0.588	9.08	5.45
	DT	0.840	5.52	3.93	0.870	5.75	4.24
	GB	0.935	3.93	2.95	0.920	4.39	3.33
	RF	0.919	4.16	3.10	0.914	4.54	3.25
	NN	0.733	7.64	5.30	0.728	8.17	5.52
	SVR	0.668	9.30	7.19	0.664	9.53	7.41
Dataset D: Age + sex + historical weight	LR	0.797	7.34	5.37	0.694	8.17	5.45
	DT	0.873	5.37	4.16	0.823	6.81	4.84
	GB	0.919	4.46	3.40	0.899	4.99	3.78
	RF	0.897	4.77	3.56	0.894	5.14	3.71
	NN	0.761	6.88	4.69	0.758	7.26	4.77
	SVR	0.756	7.94	6.20	0.752	8.17	6.43
Dataset E: Age + breed + historical weight	LR	0.721	8.70	5.98	0.578	9.46	6.05
	DT	0.809	6.81	4.99	0.806	7.19	5.07
	GB	0.908	4.77	3.63	0.882	5.45	4.08
	RF	0.879	5.22	3.71	0.875	5.60	4.08
	NN	0.730	7.79	5.45	0.725	8.32	5.67
	SVR	0.616	10.14	7.94	0.613	10.29	8.09

Note: RMSE and MAE are reported in kilograms following inverse transformation of the standardised prediction errors to improve the practical interpretation of forecasting performance. Higher R^2^ values indicate better predictive performance. Lower RMSE and MAE values indicate smaller prediction errors.

**Table 12:** Accuracy assessment of three-month-ahead HB-CLW forecasting using training and testing datasets.

Dataset Configuration	Model	R^2^ Training	RMSE Training (kg)	MAE Training (kg)	R^2^ Testing	RMSE Testing (kg)	MAE Testing (kg)
Dataset A: All factors	LR	0.769	6.18	4.60	0.532	7.69	4.74
	DT	0.827	4.94	3.78	0.824	5.22	3.91
	GB	0.924	3.36	2.54	0.902	3.85	2.88
	RF	0.897	3.71	2.82	0.894	4.05	3.02
	NN	0.629	6.46	4.19	0.623	6.94	4.40
	SVR	0.708	6.87	5.36	0.704	7.01	5.56
Dataset B: Age + historical weight	LR	0.591	8.24	5.98	0.337	9.41	6.11
	DT	0.647	7.35	5.22	0.644	7.62	5.56
	GB	0.847	4.94	3.85	0.795	5.77	4.33
	RF	0.796	5.56	4.12	0.793	5.77	4.33
	NN	0.528	8.24	6.05	0.525	8.59	6.11
	SVR	0.512	8.93	7.21	0.509	9.07	7.41
Dataset C: Age + weather + historical weight	LR	0.668	7.42	5.36	0.359	9.00	5.49
	DT	0.803	5.36	4.05	0.798	5.70	4.19
	GB	0.906	3.78	2.88	0.877	4.33	3.30
	RF	0.880	4.05	3.09	0.875	4.40	3.23
	NN	0.549	7.97	6.05	0.544	8.45	6.25
	SVR	0.529	8.65	6.66	0.525	8.93	6.94
Dataset D: Age + sex + historical weight	LR	0.717	6.87	5.15	0.547	7.90	5.29
	DT	0.742	6.25	4.67	0.739	6.52	4.81
	GB	0.873	4.46	3.43	0.840	5.01	3.91
	RF	0.844	4.74	3.64	0.840	5.01	3.85
	NN	0.687	6.66	4.94	0.684	7.01	5.01
	SVR	0.691	7.07	5.56	0.688	7.21	5.70
Dataset E: Age + breed + historical weight	LR	0.593	8.24	6.05	0.335	9.41	6.11
	DT	0.659	7.28	5.29	0.655	7.55	5.42
	GB	0.848	4.94	3.85	0.798	5.70	4.33
	RF	0.799	6.04	4.12	0.799	5.70	4.26
	NN	0.533	8.17	6.05	0.527	8.65	6.25
	SVR	0.484	9.07	7.14	0.480	9.34	7.35

Note: RMSE and MAE are reported in kilograms following inverse transformation of the standardised prediction errors to improve the practical interpretation of forecasting performance. Higher R^2^ values indicate better predictive performance. Lower RMSE and MAE values indicate smaller prediction errors.

**Table 13:** Approximate prediction interval half-widths across forecasting periods.

Forecasting Period	Standardised PI Half-Width	Approximate Target SD (kg)	PI Half-Width (kg)
One Month	0.041	79.57	3.26 kg
Two Months	0.057	75.65	4.31 kg
Three Months	0.074	68.68	5.08 kg

Note: Standardised 95% interval half-widths were multiplied by full dataset target SDs. These SDs approximated unavailable fold-specific conversion factors. Total interval width equals twice the reported half-width.

**Table 14:** Feature-importance rankings derived from permutation importance analysis across forecasting periods.

Predictor	One-Month Importance	Two-Month Importance	Three-Month Importance
Age	0.41	0.39	0.37
Previous Weight	0.33	0.31	0.29
Rainfall (Combined)	0.11	0.14	0.16
Temperature (Combined)	0.09	0.11	0.13
Sex	0.04	0.03	0.03
Breed	0.02	0.02	0.02

Note: Permutation importance values for rainfall and temperature are presented as combined climatic contributions across all lag intervals. Lag-specific climatic effects are reported separately in Table 15 using SHAP analysis.

**Table 15:** SHAP importance ranking of climatic lag predictors across forecasting periods.

Predictor Variable	One-Month Forecasting	Two-Month Forecasting	Three-Month Forecasting
Current-Month Rainfall	0.041	0.062	0.071
Previous First-Month Rainfall	0.055	0.091	0.113
Previous Second-Month Rainfall	0.038	0.084	0.108
Current-Month Temperature	0.044	0.059	0.068
Previous First-Month Temperature	0.049	0.073	0.092
Previous Second-Month Temperature	0.036	0.067	0.089

**Table 16:** Residual and influence diagnostics across forecasting periods.

Forecasting Period	Mean GB Residual (kg)	MAD Below 450 kg	MAD at or Above 450 kg	Brown and Forsythe Statistic	*p*-Value	LR Influential Observations	Affected Cattle	Maximum Cook’s Distance
One month	0.08	2.85	5.62	14.73	<0.001	37	24	0.021
Two months	0.14	3.11	6.24	16.89	<0.001	34	22	0.027
Three months	0.21	3.38	7.02	19.41	<0.001	31	20	0.034

## Data Availability

The data presented in this study are available on request from the corresponding author.
